# Individual and combined effects of DNA methylation and copy number alterations on miRNA expression in breast tumors

**DOI:** 10.1186/gb-2013-14-11-r126

**Published:** 2013-11-20

**Authors:** Miriam Ragle Aure, Suvi-Katri Leivonen, Thomas Fleischer, Qian Zhu, Jens Overgaard, Jan Alsner, Trine Tramm, Riku Louhimo, Grethe I Grenaker Alnæs, Merja Perälä, Florence Busato, Nizar Touleimat, Jörg Tost, Anne-Lise Børresen-Dale, Sampsa Hautaniemi, Olga G Troyanskaya, Ole Christian Lingjærde, Kristine Kleivi Sahlberg, Vessela N Kristensen

**Affiliations:** 1 Department of Genetics, Institute for Cancer Research, Oslo University Hospital, The Norwegian Radiumhospital, 0310 Oslo, Norway; 2 The KG Jebsen Center for Breast Cancer Research, Institute of Clinical Medicine, Faculty of Medicine, University of Oslo, 0318 Oslo, Norway; 3 Department of Computer Science, Princeton University, Princeton, NJ 08540 USA; 4 Department of Experimental Clinical Oncology, Aarhus University Hospital, 8000 Aarhus, Denmark; 5 Systems Biology Laboratory, Institute of Biomedicine and Genome-Scale Biology Research Program, University of Helsinki, 00014 Helsinki, Finland; 6 Medical Biotechnology, VTT Technical Research Centre of Finland, 20521 Turku, Finland; 7 Laboratory for Epigenetics and Environment, Centre National de Génotypage, CEA - Institut de Génomique, 91000 Evry, France; 8 Lewis-Sigler Institute for Integrative Genomics, Princeton University, Princeton, NJ 08544 USA; 9 Biomedical Informatics Research Group, Department of Informatics, University of Oslo, 0316 Oslo, Norway; 10 Centre for Cancer Biomedicine, University of Oslo, 0424 Oslo, Norway; 11 Department of Research, Vestre Viken Hospital Trust, 3004 Drammen, Norway; 12 Department of Clinical Molecular Biology and Laboratory Science (EpiGen), Division of Medicine, Akershus University Hospital, 1478 Akershus, Norway

## Abstract

**Background:**

The global effect of copy number and epigenetic alterations on miRNA expression in cancer is poorly understood. In the present study, we integrate genome-wide DNA methylation, copy number and miRNA expression and identify genetic mechanisms underlying miRNA dysregulation in breast cancer.

**Results:**

We identify 70 miRNAs whose expression was associated with alterations in copy number or methylation, or both. Among these, five miRNA families are represented. Interestingly, the members of these families are encoded on different chromosomes and are complementarily altered by gain or hypomethylation across the patients. In an independent breast cancer cohort of 123 patients, 41 of the 70 miRNAs were confirmed with respect to aberration pattern and association to expression. *In vitro* functional experiments were performed in breast cancer cell lines with miRNA mimics to evaluate the phenotype of the replicated miRNAs. *let-7e-3p*, which in tumors is found associated with hypermethylation, is shown to induce apoptosis and reduce cell viability, and low *let-7e-3p* expression is associated with poorer prognosis. The overexpression of three other miRNAs associated with copy number gain, *miR-21-3p*, *miR-148b-3p* and *miR-151a-5p*, increases proliferation of breast cancer cell lines. In addition, *miR-151a-5p* enhances the levels of phosphorylated AKT protein.

**Conclusions:**

Our data provide novel evidence of the mechanisms behind miRNA dysregulation in breast cancer. The study contributes to the understanding of how methylation and copy number alterations influence miRNA expression, emphasizing miRNA functionality through redundant encoding, and suggests novel miRNAs important in breast cancer.

## Background

MicroRNAs (miRNAs) are small, non-coding RNA molecules that regulate gene expression at a post-transcriptional level by controlling mRNA stability and translation. They are encoded either in the introns of protein-coding host genes or independently in intergenic regions [1]. The initial primary miRNA transcript can function as a polycistronic transcript giving rise to several functional miRNAs. The 18 to 24 nucleotide long mature miRNA is spliced from a hairpin structure where the two complementary strands both can function as regulators. In the mature sequence, the nucleotides 2 to 8 are crucial as they constitute the ‘seed’ sequence which is important for target recognition and binding. Similarities in the seed sequence are used to group miRNAs into families [2].

In the normal cellular context, miRNAs may act as genetic switches or fine-tuners [3]. Aberrant expression of miRNAs has frequently been reported in cancer [4-7] and miRNAs are thus suggested to play potential oncogenic or tumor-suppressive roles. In breast cancer, altered miRNA expression has been associated with, for example, estrogen receptor (ER) signaling [8-10], proliferation [4,11,12] and metastasis [13-16]. The underlying mechanisms of aberrant miRNA expression are poorly understood, but likely causes include DNA copy number aberrations, mutations, epigenetic aberrations, dysregulation of transcription factors targeting miRNAs and alterations in the miRNA biogenesis pathway [17]. A recent example of the latter was the finding that EGFR can modulate miRNA maturation in response to hypoxia [18]. One of the very first associations of miRNA deregulation with human cancer was with the discovery of deletion and subsequent down-regulation of the *MIR15* and *MIR16* genes at 13q14 in chronic lymphocytic leukemia [19]. Later, several reports have followed linking miRNA genes and their expression to genomic regions associated with gain and loss in cancer [20-22]. In a recent study, miRNAs were shown to be over-represented in copy-altered compared to copy-neutral regions in breast cancer, although the effect on miRNA expression was not incisive [23].

DNA methylation is an epigenetic modification where methyltransferases add methyl groups to cytosine residues followed by guanines (CpGs). CpG islands are CpG rich regions often found in gene promoters, and the methylation state of a CpG island often correlates with the gene expression state with hypermethylation associated with decreased expression and hypomethylation with increased expression [24]. Recently, aberrant DNA methylation of miRNA genes has received attention and been identified as an emerging mechanism of miRNA deregulation in cancer [25]. In particular, hypermethylation of miRNA promoters has been reported, but also hypomethylation has been recognized as a mechanism leading to disrupted miRNA expression in cancer [14,15,26-32].

In this study, we investigated the combined effect of copy number and methylation on miRNA expression in breast cancer. The analysis revealed complementary aberration patterns across patients and led to the discovery of novel tumor suppressing and oncogenic miRNA candidates, of which four selected miRNAs were confirmed by functional studies *in vitro*.

## Results

### Integrated analysis reveals miRNAs altered in*-cis* by copy number or DNA methylation

**Figure 1:**
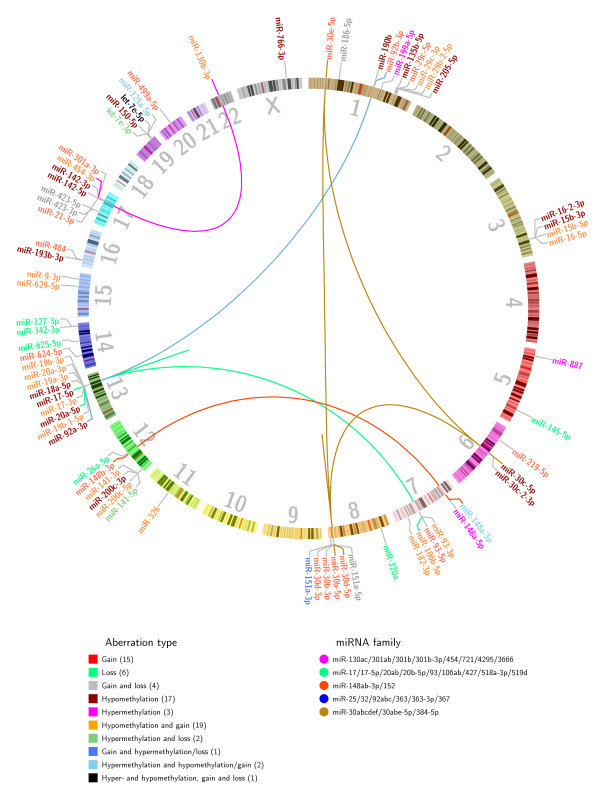
**Genomic localization of the 70 in-*cis *miRNAs.** The 70 in-*cis* miRNAs were identified using Wilcoxon rank-sum tests to identify differential miRNA expression between patients in different aberration groups. miRNAs are color coded according to main aberration type of the miRNA locus (see legend). The number in parentheses under aberration type represents the number of in-*cis* miRNAs in the given aberration category. Inter-chromosomal and intra-chromosomal lines (the latter is seen as independent lines on chromosomes 8 and 13) link in*-cis* miRNAs to other members of the same miRNA family (see legend). miRNA family annotation is taken from TargetScan (release 6.2) [2,33]. The plot was made using the Circos software package [34].

To identify potential oncogenic or tumor suppressor miRNAs, two scenarios of particular biological relevance were considered. In the first scenario, lower expression is due to loss of genetic material or hypermethylation (silencing in-*cis* effects), and in the second, higher expression is due to gain of genetic material or hypomethylation (activating in-*cis* effects). Considering 575 miRNA genomic loci (corresponding to 461 unique and detectable mature miRNAs), 70 miRNAs were classified as in-*cis* miRNAs, that is, methylation or copy number aberrations were associated with the miRNA expression level (Figure 1; Additional file 1). For silencing in-*cis* effects, we considered hypermethylation alone, loss alone, and hypermethylation and loss combined. For activating in-*cis* effects, we considered hypomethylation alone, gain alone and finally hypomethylation and gain combined. This allowed the contribution of both methylation and copy number aberrations to be captured, thus enabling the identification of the main force driving aberrant miRNA expression. Additional file 2 shows an outline of the approach.

**Figure 2:**
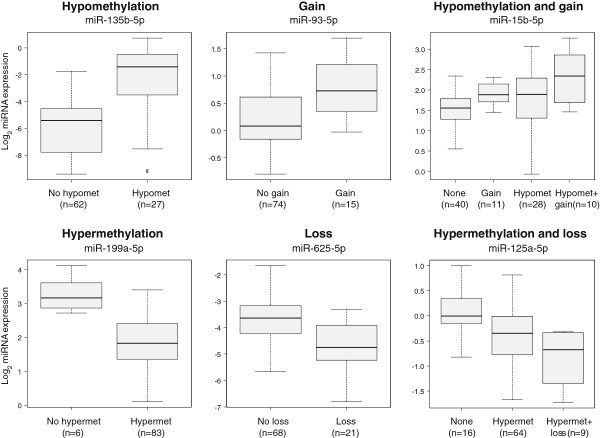
**Examples of in-*cis *miRNAs.** Six different miRNAs are depicted whose expression was associated with a certain aberration type in the breast cancer discovery cohort. The boxplots show the miRNA expression among aberration groups and the number in parentheses indicate the number of patients in a given aberration group. Hypomet, hypomethylation; hypermet, hypermethylation.

Among the 70 in*-cis* miRNAs, 24 were associated mainly with copy number aberrations, 22 mainly with methylation aberrations and 24 miRNAs with a combination of copy number and methylation aberrations (see Additional file 1 for details). Figure 2 shows examples of identified in-*cis* miRNAs from the different aberration categories. A total of 59 in-*cis* miRNAs had an association between hypomethylation or gain and increased expression, and 19 had an association between hypermethylation or loss and decreased expression. Eight miRNA loci showed both silencing and activating in-*cis* effects.

In-*cis* miRNAs were most frequently found to reside on chromosome 1 (14%), 8 (10%), 13 (13%) and 17 (10%), all of which are commonly associated with aberrations in breast cancer [35]. The average Spearman correlation between copy number and expression for miRNAs that were mainly copy number driven was 0.44 (ranging from 0.22 to 0.65). For the miRNAs mainly driven by methylation aberrations the average Spearman correlation between methylation status and expression was -0.50 (ranging from -0.65 to -0.31; Additional file 1).

### Complementary aberration pattern of miRNAs with identical seed sequence

Among the 70 in-*cis* miRNAs, five families were identified encompassing a total of 13 in-*cis* miRNAs (Table 1). All members of each family have identical seed sequence, but are frequently encoded on different chromosomes (Figure 1). Due to the identical seed sequence, miRNAs in the same family may share common target genes [2]. Several of the tumors had an aberration of at least one of the miRNAs in each family (Additional file 3), showing a complementary aberration pattern for these miRNAs in breast cancer. For example, the in*-cis* miRNAs miR-30b-5p/miR-30d-5p, miR-30c-5p and miR-30e-5p are encoded on chromosomes 8, 6, and 1, respectively. Several of the tumor samples were found to have gain or hypomethylation of at least one of these miRNA loci (Additional file 3). Similarly, miR-92a-3p and miR-92b-3p, encoded on chromosomes 13 and 1, respectively, were associated with hypomethylation and gain leading to increased expression. In addition, miR-106b-5p (chromosome 7), miR-17-5p and miR-20a-5p (both on chromosome 13) were associated with hypomethylation and gain. Interestingly, these three latter miRNAs, in addition to miR-92a-3p, are part of the paralogous miR-17-92 and miR-106b-25 clusters that have been found to be upregulated and to have an oncogenic function in several cancer types, including breast cancer [36,37]. This complementary mode of aberrations further suggests that these miRNAs play an important oncogenic role in breast cancer, and shows that both hypomethylation and gain are mechanisms that increase their expression across the patients.

**Table 1:** **In**-***cis *miRNA families and family members**

**miRNA family**^**a**^	**miRNA**	**MIMAT**	**Chromosomal location (hg19)**	**Mature sequence (5′-3′ direction)**^**b**^	**Aberration**^**c**^
miR-148ab-3p/152	miR-148a-3p	MIMAT0000243	7p15.2	U**CAGUGCA**CUACAGAACUUUGU	**Hypermethylated**/hypogain
	miR-148b-3p	MIMAT0000759	12q13.13	U**CAGUGCA**UCACAGAACUUUGU	**Gain**
miR-17/17-5p/20ab/20b-5p/93/106ab/427/518a-3p/519d	miR-106b-5p	MIMAT0000680	7q22.1	U**AAAGUGC**UGACAGUGCAGAU	**Hypogain**
	miR-17-5p	MIMAT0000070	13q31.3	C**AAAGUGC**UUACAGUGCAGGUAG	**Hypomethylated**/hypogain
	miR-20a-5p	MIMAT0000075	13q31.3	U**AAAGUGC**UUAUAGUGCAGGUAG	**Hypomethylated**/hypogain
miR-130ac/301ab/301b/301b-3p/454/721/4295/3666	miR-130b-3p	MIMAT0000691	22q11.21	C**AGUGCAA**UGAUGAAAGGGCAU	**Hypogain**
	miR-454-3p	MIMAT0003885	17q22	U**AGUGCAA**UAUUGCUUAUAGGGU	**Hypogain**
miR-30abcdef/30abe-5p/384-5p	miR-30b-5p	MIMAT0000420	8q24.22	U**GUAAACA**UCCUACACUCAGCU	**Gain**
	miR-30c-5p	MIMAT0000244	6q13	U**GUAAACA**UCCUACACUCUCAGC	**Hypomethylated**
	miR-30d-5p	MIMAT0000245	8q24.22	U**GUAAACA**UCCCCGACUGGAAG	**Gain**/Hypogain
	miR-30e-5p	MIMAT0000692	1p34.2	U**GUAAACA**UCCUUGACUGGAAG	**Gain**
miR-25/32/92abc/363/363-3p/367	miR-92a-3p	MIMAT0000092	13q31.1	U**AUUGCAC**UUGUCCCGGCCUGU	**Hypomethylated**/hypogain
	miR-92b-3p	MIMAT0003218	1q22	U**AUUGCAC**UCGUCCCGGCCUCC	**Gain**/Hypogain

^a^From TargetScan (release 6.2) [2,33].

^b^From miRBase (release 18) [38]. Nucleotides in bold highlight the seed sequence.

^c^According to Wilcoxon rank-sum tests. Aberrations highlighted in bold indicate main aberration type. Hypogain, significant in tests grouping samples with hypomethylation and/or gain.

### Replication of the in*-cis* miRNAs in an independent breast cancer cohort

In order to validate the 70 identified in-*cis* miRNAs, miRNA expression and copy number data from an independent breast cancer cohort consisting of 123 breast cancer patients were analyzed. In addition, 26 in-*cis* miRNAs were selected for methylation aberration assessment by pyrosequencing. Of the 70 in-*cis* miRNAs, 41 were consistently associated with similar activating or silencing in-*cis* effects in the replication cohort as in the discovery cohort (Table 2). Interestingly, two of the five miRNA families with members residing on different chromosomes were represented among the replicated miRNAs; miR-130b-3p/miR-454-3p on chromosomes 22 and 17, respectively, and miR-30c-5p/miR-30d-5p on chromosomes 6 and 8, respectively.

**Table 2:** **In-*cis *** **miRNAs confirmed in the replication cohort**

**miRNA**	**MIMAT id**	**Chromosomal location (hg19)**	**Replicated aberration type**
let-7e-3p	MIMAT0004485	19q13.41	Hypermethylation
miR-125a-5p	MIMAT0000443	19q13.41	Hypermethylation
miR-130b-3p	MIMAT0000691	22q11.21	Gain
miR-135b-5p	MIMAT0000758	1q32.1	Hypomethylation
miR-141-3p	MIMAT0000432	12p13.31	Gain
miR-142-3p	MIMAT0000434	17q22	Hypomethylation
miR-142-5p	MIMAT0000433	17q22	Hypomethylation
miR-148a-3p	MIMAT0000243	7p15.2	Hypermethylation
miR-148a-5p	MIMAT0004549	7p15.2	Hypermethylation
miR-148b-3p	MIMAT0000759	12q13.13	Gain
miR-151a-3p	MIMAT0000757	8q24.3	Gain
miR-151a-5p	MIMAT0004697	8q24.3	Gain
miR-15b-3p	MIMAT0004586	3q25.33	Gain and hypomethylation
miR-16-2-3p	MIMAT0004518	3q25.33	Hypomethylation
miR-17-3p	MIMAT0000071	13q31.3	Hypomethylation
miR-17-5p	MIMAT0000070	13q31.3	Hypomethylation
miR-182-3p	MIMAT0000260	7q32.2	Gain
miR-186-5p	MIMAT0000456	1p31.1	Gain and loss
miR-190b	MIMAT0004929	1q21.3	Hypomethylation
miR-193b-3p	MIMAT0002819	16p13.12	Gain
miR-19a-3p	MIMAT0000073	13q31.3	Hypomethylation
miR-19b-3p	MIMAT0000074	13q31.3	Hypomethylation
miR-200c-3p	MIMAT0000617	12p13.31	Gain and hypomethylation
miR-200c-5p	MIMAT0004657	12p13.31	Gain
miR-205-5p	MIMAT0000266	1q32.2	Hypomethylation
miR-21-3p	MIMAT0004494	17q23.1	Gain
miR-219-5p	MIMAT0000276	6p21.32	Gain
miR-29b-2-5p	MIMAT0004515	1q32.2	Gain
miR-301a-3p	MIMAT0000688	17q22	Gain
miR-30b-3p	MIMAT0004589	8q24.22	Gain
miR-30c-2-3p	MIMAT0004550	6q13	Hypomethylation
miR-30c-5p	MIMAT0000244	6q13	Hypomethylation
miR-30d-3p	MIMAT0004551	8q24.22	Gain
miR-30d-5p	MIMAT0000245	8q24.22	Gain
miR-423-3p	MIMAT0001340	17q11.2	Gain
miR-423-5p	MIMAT0004748	17q11.2	Gain
miR-454-3p	MIMAT0003885	17q22	Gain
miR-484	MIMAT0002174	16p13.11	Gain
miR-93-3p	MIMAT0004509	7q22.1	Gain
miR-93-5p	MIMAT0000093	7q22.1	Gain
miR-9-3p	MIMAT0000442	15q26.1	Gain

Of the 41 replicated in-*cis* miRNAs, 25 were subject to similar copy number aberration in both the replication and discovery cohort. These 25 copy number-replicated miRNAs were among those with the highest copy number-expression correlation in both cohorts when considering all in*-cis* miRNAs (Additional file 4a). miR-186-5p was the only replicated miRNA associated with copy number loss and decreased expression. Interestingly, it was also associated with upregulation in samples with gain, and thus one of the in*-cis* miRNAs with an ambiguous aberration pattern.

Furthermore, in the replication cohort, 14 miRNAs were found associated with hypomethylation and increased expression, and four miRNAs were associated with hypermethylation and decreased expression, in agreement with those observed in the discovery cohort. Two miRNAs, miR-15b-3p and miR-200c-3p, were associated with both gain and hypomethylation. The Spearman correlation between methylation status and miRNA expression in the discovery and replication cohorts was 0.56 (Additional file 4b).

### Replicated in*-cis* miRNAs and associations with clinical parameters

To investigate whether the expression of the replicated in*-cis* miRNAs was significantly associated with clinical or molecular subgroups in breast cancer, Wilcoxon rank-sum tests were used. A false discovery rate (FDR) adjusted threshold of *P* < 0.05 was considered as statistically significant. The parameters investigated were *TP53* mutation status, histological grade, molecular subtypes (luminal versus basal-like), ER and human epidermal growth factor receptor 2 (HER2) status. The in-*cis* miRNAs consistently associated with clinical parameters in both cohorts and the associated *P*-values are shown in Table 3 (those associated with clinical parameters in either one of the cohorts are shown in Additional file 5). miR-19a-3p, which was associated with hypomethylation, was found up-regulated in *TP53* mutated, ER-negative and basal-like samples. miR-93-5p and miR-30c-2-3p associated with gain and hypomethylation, respectively, were found to be inversely associated with grade; miR-93-5p expression increasing with increasing grade, and miR-30c-2-3p expression decreasing with increasing grade. Interestingly, miR-93-5p expression was previously found positively associated with increasing grade, and mir-30a-3p/5p expression was inversely associated with grade [39]. As expected, there was considerable overlap between miRNAs differentially expressed between the luminal and basal-like subtypes and the ER-positive and -negative tumors. For example, miR-125a-5p and miR-190b showed increased expression in luminal and ER-positive tumors compared to basal-like and ER-negative tumors, whereas miR-9-3p, miR-17-5p and miR-19a-3p showed the opposite expression pattern (Table 3).

**Table 3:** **Replicated in-*cis *** **miRNAs and associations with clinical parameters**

	**miRNA**	**Chromosomal location (hg19)**	**Direction**	**Associated miRNA aberration**	***P*-value (FDR-corrected, discovery cohort)**	***P*-value (FDR-corrected, replication cohort)**
***TP53 *** **status (wild-type versus mutant)**	miR-19a-3p	13q31.3	Up in *TP53* mutated samples	Hypomethylation	7.5E-03	9.1E-03
**Grade (1, 2 and 3)**	miR-30c-2-3p	6q13	Decreasing with grade	Hypomethylation	1.7E-02	2.8E-02
	miR-93-5p	7q22.1	Increasing with grade	Gain	4.4E-03	4.2E-02
**ER status (positive (+) versus negative (-))**	miR-9-3p	15q26.1	Down in ER+/up in ER-	Gain	9.0E-04	4.1E-03
	miR-17-5p	13q31.3	Down in ER+/up in ER-	Hypomethylation	1.5E-02	2.9E-03
	miR-19a-3p	13q31.3	Down in ER+/up in ER-	Hypomethylation	2.9E-02	2.1E-05
	miR-93-5p	7q22.1	Down in ER+/up in ER-	Gain	3.8E-02	2.7E-02
	miR-130b-3p	22q11.21	Down in ER+/up in ER-	Gain	3.5E-02	5.0E-02
	miR-30c-2-3p	6q13	Up in ER+/down in ER-	Hypomethylation	4.9E-02	2.4E-02
	miR-125a-5p	19q13.41	Up in ER+/down in ER-	Hypermethylation	1.1E-02	3.1E-03
	miR-190b	1q21.3	Up in ER+/down in ER-	Hypomethylation	3.5E-07	3.0E-02
**Luminal versus basal-like subtype**	miR-125a-5p	19q13.41	Up in luminal/down in basal-like	Hypermethylation	6.4E-03	1.3E-03
	miR-148b-3p	12q13.13	Up in luminal/down in basal-like	Gain	1.6E-05	1.0E-02
	miR-190b	1q21.3	Up in luminal/down in basal-like	Hypomethylation	1.6E-05	1.7E-04
	miR-193b-3p	16p13.12	Up in luminal/down in basal-like	Gain	2.5E-04	4.0E-06
	let-7e-3p	19q13.41	Up in luminal/down in basal-like	Hypermethylation	1.5E-02	8.3E-03
	miR-9-3p	15q26.1	Down in luminal/up in basal-like	Gain	2.4E-05	4.9E-03
	miR-17-3p	13q31.3	Down in luminal/up in basal-like	Hypomethylation	2.5E-05	1.9E-04
	miR-17-5p	13q31.3	Down in luminal/up in basal-like	Hypomethylation	1.8E-04	1.2E-04
	miR-19b-3p	13q31.3	Down in luminal/up in basal-like	Hypomethylation	2.8E-05	4.0E-06
	miR-19a-3p	13q31.3	Down in luminal/up in basal-like	Hypomethylation	1.4E-04	4.0E-06
	miR-135b-5p	1q32.1	Down in luminal/up in basal-like	Hypomethylation	1.6E-05	2.8E-03
	miR-142-5p	17q22	Down in luminal/up in basal-like	Hypomethylation	8.6E-03	1.4E-02

### *In vitro* functional studies of the candidate miRNAs reveal importance for cancer cell survival

To study the functional significance of the replicated in-*cis* miRNAs, we performed miRNA gain-of-function studies in three breast cancer cell lines using miRNA mimics. The KPL-4 and JIMT-1 cell lines used are ER-negative, while MCF-7 is an ER-positive cell line. Cell viability, proliferation, phosphorylated AKT levels (p-AKT) and apoptosis (cleaved poly (ADP-ribose) polymerase (cPARP)) were used as endpoints. Of the 41 in-*cis* miRNAs tested, four miRNAs in particular, miR-21-3p, miR-148b-3p, miR-151a-5p and let-7e-3p, showed significant and consistent associations between aberration patterns in the breast cancer tumors and phenotypic effects when overexpressed in cell lines.

**Figure 3:**
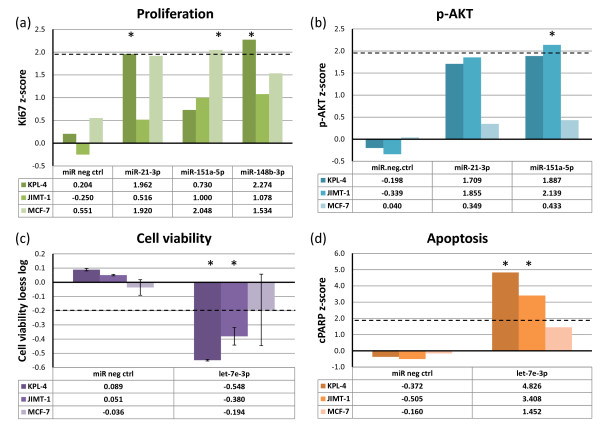
**In*-cis *miRNAs show functional effects when overexpressed.** Breast cancer cell lines were transfected with miRNA mimics (20 nM) and assayed for **(a)** cell proliferation (Ki67), **(b)** phosphorylated AKT (p-AKT) levels, **(c)** cell viability and **(d)** apoptosis (cleaved PARP (cPARP)), 72 hours after transfection. The dashed lines indicate cut-off points that were considered significant (see Materials and methods). Asterisks denote significant effects. The data for cell viability are from two replicate experiments with error bars showing standard deviations.

The coding sequence of miR-21-3p on chromosome 17q23.1 was associated with gain and subsequent higher expression in >35% of the patient samples in both cohorts (Additional file 6a; *P* < 0.001 for both cohorts). Overexpression of miR-21-3p resulted in increased proliferation in the breast cancer cell lines KPL-4 and MCF-7, as measured by increased levels of the proliferation marker Ki67 (Figure 3a). In addition, the levels of phosphorylated AKT protein were increased as a result of miR-21-3p overexpression in the KPL-4 and JIMT-1 cell lines (although not significant), indicating enhanced activity of the AKT pathway (Figure 3b).

Patients with miR-151a-5p gain showed significantly higher expression of this miRNA (Additional file 6b; *P* < 0.001 and *P* = 0.001 in the discovery and replication cohorts, respectively). Proliferation was significantly increased when overexpressing miR-151a-5p in the MCF-7 cell line, and increased proliferation was also evident in the KPL-4 and JIMT-1 cell lines (Figure 3a). Furthermore, miR-151a-5p overexpression led to an increase of p-AKT levels in the JIMT-1 and KPL-4 cell lines (Figure 3b). Thus, it is possible that miR-151a-5p directly or indirectly activates the AKT pathway and subsequently cell proliferation.

**Figure 4:**
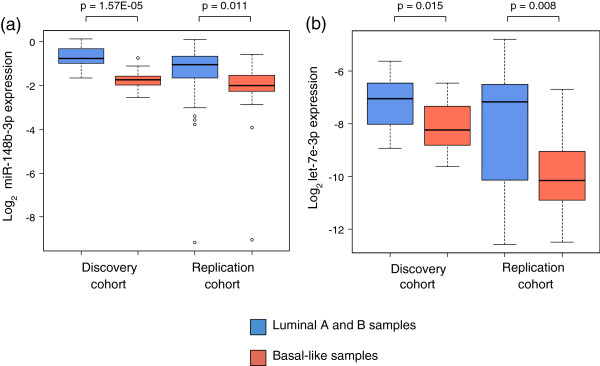
**miRNA expression in luminal and basal-like patients.** miRNA expression when patients were divided into luminal and basal-like subgroups in the discovery and replication cohort. **(a)** miR-148b-3p expression, **(b)** let-7e-3p expression. The *P*-values are from Wilcoxon rank-sum tests.

miR-148b-3p was associated with significantly higher expression in patients with gain (Additional file 6c; *P* = 0.002 and *P* = 0.008 in the discovery and replication cohorts, respectively). Overexpression of miR-148b-3p significantly increased cell proliferation in the KPL-4 cell line, and an increased effect on proliferation was also seen in the MCF-7 and JIMT-1 cell lines (Figure 3a). Interestingly, miR-148b-3p was one of the miRNAs differentially expressed between luminal and basal-like subtypes (Table 3), showing higher expression in luminal samples compared to basal-like samples (Figure 4a).

**Figure 5:**
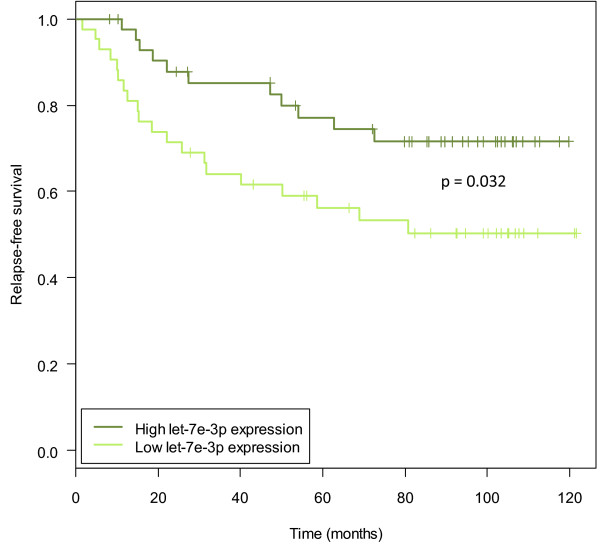
**Kaplan-Meier curves showing relapse-free survival when dividing samples into high and low let-7e-3p expression groups.** The ‘low’ group denotes samples with expression below the median (n = 43) and the ‘high’ group contains samples with expression above the median (n = 43). The *P*-value is from a log-rank test. The plot shows survival data of the discovery cohort.

Hypermethylation of the let-7e-3p miRNA promoter on chromosome 19q13.41 was associated with decreased let-7e-3p expression (Additional file 6d; *P* = 0.002 and *P* = 0.001 in the discovery and replication cohorts, respectively). Overexpression of let-7e-3p decreased cell viability (Figure 3c), and induced apoptosis (Figure 3d) in the KPL-4 and JIMT-1 cell lines. let-7e-3p was higher expressed in luminal samples compared to basal-like samples (Figure 4b). It was also found significantly more highly expressed in ER-positive compared to ER-negative samples in the replication cohort (Additional file 5). Furthermore, in the discovery cohort, low expression of let-7e-3p was associated with poorer prognosis (Figure 5; log-rank test *P* = 0.032). Statistical significance was not found in the replication cohort (*P* = 0.170).

### Gene expression (anti-)correlated to the candidate miRNAs

To decipher the biological processes the four candidate miRNAs might regulate, we explored the highest correlated genes associated with these miRNAs. For this task, a miRNA-mRNA expression correlation analysis calculating the Spearman correlation for each miRNA to all genes was performed. Both positive (Spearman’s rho >0.3) and negative (Spearman’s rho < -0.3) correlations were considered, but only those consistently correlated in the two cohorts. The numbers of correlated genes were 277 for miR-148b-3p, 53 for let-7e-3p, 32 for miR-21-3p, and 22 genes for miR-151a-5p (Additional file 7).

Two approaches were used to characterize the genes correlated with the candidate miRNAs. First, Ingenuity Pathway Analysis (IPA) was used to identify networks associated with these genes. Then, for each candidate miRNA, the positively or negatively correlated genes were expanded based on co-expression using the Search-based Exploration of Expression Compendium (SEEK), and the set of co-expressed genes thus identified was used for functional enrichment analysis. This resulted in Gene Ontology biological process terms found enriched among the genes co-expressed with the genes correlated to the miRNAs. Additional file 8 lists the SEEK results.

miR-148b-3p expression was correlated to several genes associated with cancer-related biological functions, such as growth, proliferation and cell death, according to IPA. For example, *EGFR*, *ERBB3*, *FAS* and *CCND2* expression was correlated with miR-148b-3p expression (Additional file 9a). The SEEK-identified genes co-expressed with the negatively correlated genes showed enrichment of processes related to the Wnt pathway and motility. The genes co-expressed with the positively correlated genes were enriched for processes related to the electron transport chain and ATP synthesis.

For let-7e-3p, cancer-associated genes such as *MAPK1* and *EZH2* were among the negatively correlated genes, and *PURA*, *ERCC1* and *MAPT* were among the positively correlated ones (Additional file 9b). The top networks according to IPA were cell cycle, gene expression, cancer, gastrointestinal disease and hepatic system disease. The SEEK-identified genes co-expressed with the negatively correlated genes were enriched for various cell cycle- and mitosis-related biological processes. This is consistent with the here demonstrated ability of let-7e-3p to induce apoptosis and inhibit cell viability when overexpressed in breast cancer cells.

There were mainly positively correlated genes associated with the expression of miR-21-3p, and the biological functions of the related networks were both disease and motility related according to IPA (Additional file 9c). Using SEEK, migration was consistently identified when considering the positively correlated genes, in addition to various vasculature-related processes.

miR-151a-5p expression was correlated to 22 genes in the discovery and replication cohort, however, including only one negatively correlated gene (*PAPLN*). No network was found for the correlated genes according to IPA. SEEK identified processes related to motility and lipid kinase activity associated with the positively correlated genes. One of these genes, *PTK2*, is associated with the AKT pathway [40]. These findings could be coupled to the here demonstrated increased proliferation and elevated p-AKT levels observed when overexpressing miR-151a-5p in cell lines (Figure 3).

## Discussion

The contribution of miRNA deregulation to cancer development and progression has become increasingly evident in recent years. However, a complete understanding of the causes of deregulation is still lacking. The aim of this study was to get a deeper insight into the underlying mechanisms of miRNA deregulation in breast cancer by integrating different layers of data from both the DNA and RNA levels. By considering two well-known mechanisms - DNA methylation and copy number alterations - we wanted to identify miRNAs affected by such alterations at the genomic and epigenetic levels that were further reflected in miRNA expression. This could indicate selection for activation or inactivation in the tumors. Alterations at the genomic copy number level that are subsequently reflected in RNA expression have been previously reported in cancer for miRNAs as well as other non-coding RNA species such as long non-coding RNAs and small nucleolar RNAs [41,42]. In contrast to most studies, which have focused on either copy number or methylation aberrations separately, this study sought to investigate both mechanisms simultaneously. Despite the use of different copy number platforms and technologies to assess methylation status, 41 miRNAs were consistently found associated with DNA methylation or copy number alteration and association with expression in the discovery and replication cohort. The Agilent miRNA microarrays that were used for expression profiling of the discovery and replication cohort have been shown to have a high performance in a comprehensive comparison study of different miRNA array technologies [43]. Furthermore, miRNA array expression validation by real-time PCR has previously been performed for a selection of the miRNAs in the discovery cohort, showing good correlation between array and real-time PCR (average Pearson correlation of 0.72) [4].

Among the in-*cis* miRNAs, some were shown to be dominated either by copy number or methylation alterations, but many were driven by a combination of both mechanisms across the patients. Interestingly, this dualism in aberrations has previously been observed in mRNA transcriptome data of glioblastoma and ovarian cancer [44], suggesting that it is an important mechanism in cancer. Another mechanism that may impact the role of miRNAs in cancer is somatic mutations located in the miRNA itself or in the miRNA binding site of a target mRNA. This could ultimately either strengthen or hamper the miRNA-mRNA binding and thus interfere with the miRNA regulatory role. Furthermore, with the relative high somatic mutation rate found in several cancers, including breast cancer [45], one may hypothesize that the combination of mutation and, for example, copy number alteration may occur in tumors. The large amounts of sequencing data that are being generated will shed new light on the prevalence of this in cancer.

The genomic distribution (intronic versus intergenic), the redundancy in being coded at various loci and chromosomes, and the sequence similarities of miRNAs allowing them to target the same genes are essential attributes to keep in mind when trying to understand miRNA-driven regulation in an evolutionary perspective. As one miRNA can have several targets, selection may act in an efficient manner; by altering one miRNA, the downstream effects may be vast. We were interested in mapping and categorizing aberration patterns that were seen across breast cancer tumors and causing phenotypic effects. Considering all miRNA genomic loci was therefore important. One could hypothesize that miRNAs with functional significance in cancer with more than one genomic origin could have ‘complementary’ regulation, that is, there are several genomic loci where an aberration can ‘hit’ in order to alter the expression of that particular miRNA. Indeed, for five miRNA families we found aberrations of several members located at different chromosomes that had a complementary aberration pattern across the patients in the discovery cohort. Furthermore, two of these families were consistently found in the independent replication cohort. For example, the miR-30 family had complementary aberrations in several members associated with hypomethylation and gain. Members of this family have previously been associated with gain and increased expression [39], and found up-regulated in ER-positive breast cancer samples [5]. In this study, miR-30c-2-3p was found consistently upregulated in ER-positive compared to ER-negative tumors. Interestingly, although associated with hypomethylation and gain resulting in upregulation, miRNAs in this family have previously also been suggested to play a tumor suppressor role; ectopic expression of miR-30 in breast-tumor initiating cells inhibited their self-renewal capacity and induced apoptosis [46]. In addition, miR-30 family members have been shown to be important for inducing cellular senescence by targeting the oncogenic transcription factor *MYBL2* in cervical carcinoma cell lines [47]. Thus, the miR-30 family miRNAs may play dualistic roles in cancer.

Although often residing on different chromosomes, miRNA members of the same family have likely arisen from ancient gene duplication events [48], and their regulatory regions may be conserved between families. Thus, members of the same family may share transcription factors. For example, the miR-17 in-*cis* family that in this study was found associated with gain and hypomethylation of members encoded on chromosomes 7 and 13, has previously been shown to be a target of the c-Myc transcription factor [49,50]. Hence, the sharing of transcriptional control elements is another mechanism for regulation of miRNA family members.

An example of miRNA redundancy in the genome is miR-125a-5p and miR-125b. The former miRNA resides on chromosome 19 and was associated with hypermethylation in both the discovery and replication cohort. miR-125b, which is encoded on chromosome 11 and 21, was previously reported as hypermethylated in breast cancer and suggested to function as a tumor suppressor [31]. miR-125a-5p and miR-125b have identical seed sequence and may thus target some of the same genes. Interestingly, Scott *et al.*[51] showed that *ERBB2* and *ERBB3* were targeted by miR-125a and miR-125b. Another validated target gene of miR-125b, *ETS1*, is a proto-oncogene [31], shown to cooperate with mutant p53 to selectively regulate promoters [52].

### In-*cis* miRNAs previously associated with cancer

Several of the replicated in*-cis* miRNAs have previously been associated with breast cancer, such as miR-93-5p [37], miR-193b-3p [9], miR-301a-3p [11] and miR-423-3p/-5p [53,54]. In addition, miRNAs found differentially expressed between ER status and molecular subtype have previously been identified [4,39]. Interestingly, miR-93-5p, which was up-regulated in ER-negative compared to ER-positive tumors, has been shown to downregulate the ER protein and inhibit estrogen-induced growth of breast cancer cell lines [9]. Two of the miRNAs associated with methylation aberrations in this study confirmed previous findings in cancer, such as hypermethylation of miR-148a-3p in breast cancer [14] and miR-199a-5p in testicular cancer [26]. In two recent studies, the role of miRNA expression during breast cancer progression was assessed, suggesting that deregulation of miRNA expression is an early event seen already during transition from normal to benign lesions [55,56]. Among the miRNAs associated with gain and hypomethylation in our study, 10 were reported upregulated in malignant compared to normal breast tissue in the latter studies: miR-15b-5p, miR-17-5p, miR-19b-3p, miR-20a-5p, miR-30d-5p, miR-106b-5p, miR-130b-3p, miR-141-3p, miR-200c-3p and miR-766-3p [55,56]. Furthermore, two miRNAs, miR-145-5p and miR-199a-5p, associated with hypermethylation and loss, were reported downregulated in malignant compared to normal tissue [55].

### Four candidate miRNAs showing functional effects

Four in-*cis* miRNAs, let-7e-3p, miR-21-3p, miR-151a-5p and miR-148b-3p, associated with *in vivo* aberrations, showed significant effects *in vitro* after functional experiments. The three former miRNAs have, to our knowledge, not been previously associated with breast cancer. Interestingly, both let-7e-3p and miR-21-3p are so-called ‘star’-miRNAs (that is, let-7e* and miR-21*). This annotation was previously used in the miRNA nomenclature to denote the miRNA sequence in the miRNA duplex that had the lowest expression and was thought to be degraded during strand selection, and thus not functional. However, it was later shown that the strand selection process is highly regulated, showing tissue-/cell-/condition-specific modulation [57]. Furthermore, the ratio of the expressed molecules from each strand varies from both strands being expressed at comparable levels to only one being expressed [57]. Though the two mature miRNAs originating from the same duplex have different target genes, they may function in a cooperative manner contributing to post-transcriptional gene silencing when co-expressed [57].

The candidate miRNAs let-7e-3p and miR-21-3p were interesting as they have been much less described than their frequently cancer-associated -5p counterparts. miR-21-5p (miR-21) has been assigned an oncogenic role and associated with overexpression in various cancer types. In breast cancer, miR-21-5p overexpression has been linked to advanced stage, metastasis and poor prognosis [58]. The *MIR21* gene resides on chromosome 17q23.1, and miR-21-5p overexpression was in a previous study linked to amplification of this genomic region [59]. Our functional data suggest that miR-21-3p may play an oncogenic role in breast cancer, in addition to its well-annotated miR-21-5p counterpart. Previous studies have shown that miR-21-5p targets *PTEN*, which further leads to increased p-AKT levels [60,61]. As miR-21-3p overexpression in this study also showed an increase in p-AKT levels, this may implicate that miR-21-5p/3p originating from the same precursor could have a coordinate way of operating.

The let-7 family of miRNAs is evolutionarily conserved across animal species, and humans have 10 mature let-7 family sequences, produced from 13 precursor sequences encoded on several chromosomes [62]. Several let-7 family members have been reported as down-regulated and thus annotated as tumor suppressor miRNAs in various cancer types [6,63,64]. In this study, we describe hypermethylation of the *MIRLET7E* promoter region associated with decreased expression of let-7e-3p. It has been previously shown that *MIRLET7E* is epigenetically repressed by lysine (K)-specific demethylase 5B (*JARID1B*) in breast cancer cell lines [12]. JARID1B is a transcriptional repressor harboring histone demethylase activity. As cyclin D1 is a target of let-7e-5p, the repression of let-7e-5p expression by JARID1B was suggested to stimulate tumor cell proliferation by allowing cyclin D1 expression and hence cell cycle progression [12]. Our functional experiments showed that overexpression of let-7e-3p had a strong positive effect on apoptosis and a strong negative effect on cell viability in the ER-negative cell lines. In the ER-positive cell line MCF-7, the same tendency was seen, although less pronounced. Interestingly, let-7e-3p was found significantly downregulated in basal-like compared to luminal samples in both cohorts tested. Thus, the stronger phenotypic effects observed in the ER-negative cell lines may be in agreement with most of the basal-like samples also being ER negative. Furthermore, low let-7e-3p expression was associated with a worse prognosis, and the network analysis linked let-7e-3p to cancer-related genes. Altogether, this suggests that let-7e-3p may also act as a tumor suppressor in breast cancer, perhaps in particular for ER-negative or basal-like tumors. To our knowledge this is the first report linking this let-7e family member to a tumor suppressor function.

miR-151a-5p is located on 8q24.3, a genomic area frequently associated with gain in breast cancer [35,65] and other cancer types [66,67]. We found high expression of miR-151a-5p associated with gain, and functional experiments showed that overexpression induced cell proliferation and also the levels of p-AKT. Altogether, this may suggest a tumorigenic role for miR-151a-5p. This miRNA has previously been linked to gain and subsequent up-regulation causing cell migration and invasion in hepatocellular carcinoma by targeting Rho GDP dissociation inhibitor alpha (*RhoGDIA*), which is a putative metastasis suppressor [21].

miR-148b-3p is encoded on chromosome 12q13.13 and was in this study associated with gain and increased expression. We showed that overexpression of miR-148b-3p increased proliferation of breast cancer cell lines. Consistently, the correlation analysis revealed both positively and negatively correlated genes associated with cell growth and proliferation. miR-148b-3p has been shown to target the methyltransferase *DNMT3b*[68]. Interestingly, this miRNA was recently reported upregulated in the blood plasma of breast cancer patients and suggested as an early detection marker [69]. However, in another study, miR-148b was found downregulated in aggressive breast tumors and suggested to inhibit progression [70]. This underlines the potential context-dependent roles of miRNAs, and was further manifested by the fact that the family member miR-148a was found associated with hypermethylation and decreased expression in both the discovery and replication cohort. Noteworthy, miR-148a was previously identified as an epigenetically silenced miRNA associated with metastasis that was reactivated when cells derived from lymph node metastases were treated with the demethylating agent 5-*aza*-2'-deoxycytidine [14]. Further functional studies are warranted to characterize the potential oncogenic role of miR-148b-3p in breast cancer.

## Conclusion

We here describe the effect of DNA methylation and copy number alterations on miRNA expression in breast cancer. Using two independent cohorts of primary breast cancer patients, we show that these mechanisms affect the expression of miRNAs. Adding to the complexity is the redundant encoding of miRNA family members, which should be taken into account to understand miRNA functionality in cancer. Altogether, this study portrays underlying mechanisms of miRNA deregulation that may contribute to breast cancer development and progression.

## Materials and methods

All computational analyses were performed in R v.2.13.0 [71] unless otherwise specified. The miRNA copy number, methylation and expression data analyzed in this work are available in Additional file 10 for the discovery cohort and Additional file 11 for the replication cohort. All experimental methods performed are in compliance with the Helsinki Declaration.

### Patient materials

Included in this study were primary breast carcinoma samples from 89 patients in the MicMa cohort [72] with data on miRNA expression, methylation, copy number and mRNA expression. All samples were fresh frozen and contained at least 40% tumor cells. The majority of the tumor specimens represent tumor size T1/T2, node status N0/N1, and histological grade 2 or 3. Tumor DNA was extracted using an ABI 341 Nucleic Acid Purification System (Applied Biosystems, Foster City, CA, USA) according to the manufacturer’s protocol. Tumor RNA was isolated using TRIzol reagent (Invitrogen, Carlsbad, CA, USA) as previously described [73]. The molecular subtype classification based on mRNA expression and the clinical information is described in [74]. The study was approved by the Norwegian Regional Committee for Medical Research Ethics, Health region II (reference number S-97103), and patients have given written consent for the use of material for research purposes.

A total of 123 primary breast carcinoma samples from the Danish Breast Cancer Cooperative Group (‘DBCG’) 82 b and c trial cohort [75-77] were obtained after total mastectomy surgery and were used for the purpose of replication. This study comprises a collection of tumor tissues from 3,083 high-risk Danish breast cancer patients diagnosed in the period 1982 to 1990 [75-77]. Total mastectomy with partial axillary dissection was performed on all women, and a median of seven lymph nodes was removed from the axilla. RNA was extracted using the Qiagen Midi kit Extraction column procedure (Qiagen GmbH, Hilden, Germany). The study of the DBCG82bc cohort has been approved by the Regional Ethical Committee (Journal number 20030263).

Normal breast tissue samples were available from 17 women that had undergone mammoplastic reduction at Colosseumklinikken, Oslo, Norway [78]. These were used as a reference when calling hyper- and hypomethylation (see below).

### miRNA expression profiling

The miRNA expression profiling for the discovery and replication cohort was performed using the 8x15k ‘Human miRNA Microarray Kit (V2)’ with design id 019118 from Agilent (Agilent Technologies, Santa Clara, CA, USA). In brief, 100 ng total RNA was dephosphorylated, labeled and hybridized for 20 hours, following the manufacturer’s protocol. Scanning was performed on Agilent Scanner G2565A, signals were extracted using Feature Extraction v9.5 and the subsequent data processing was performed using the GeneSpring software v12.0 (Agilent Technologies). As the miRNA expression for the discovery cohort was performed in duplicates per sample (on different arrays and time points), the miRNA signal intensities were averaged for replicate samples. For both the discovery and replication cohort the miRNA signal intensities were log_2_-transformed and for each sample, the 90th percentile was calculated across all miRNAs and subtracted from the miRNA expression. miRNAs that were detected in less than 10% of the samples were excluded. In the discovery cohort this resulted in 461 unique mature miRNAs. When a mature miRNA is encoded several places in the genome, the genomic origin of a transcript cannot be directly inferred from miRNA expression microarrays (unless the pre-miRNAs are detected). To account for this, we constructed an expanded 575 × 89 miRNA expression matrix that represented all the 575 miRNA genomic loci of the expressed 461 mature miRNAs using an annotation file from Agilent eArray. All subsequent analyses were based on this expanded matrix (Additional file 10). The miRNA expression data for the discovery cohort were published in [4] and have been submitted to the Gene Expression Omnibus (GEO) with accession number GSE19536. The miRNA expression data for the replication cohort have been submitted to GEO with accession number GSE46934.

### DNA methylation analysis

#### Microarray

The Infinium HumanMethylation450 BeadChip microarrays (Illumina, San Diego, CA, USA) were used to assay the 89 breast carcinomas in the discovery cohort and 17 normal breast samples from mammoplastic reduction. The samples were analyzed according to the manufacturer's protocol and normalized using a subset quantile normalization (SQN) approach based on the functional annotation of probes [79]. Probes potentially containing genetic variation with a minor allele frequency >5% in the population of European origin (CEU, 1000 Genomes Project) within the 20 bp of the 3’ probe sequence as well as the nucleotides N + 1 and N + 2 of the target sequence were omitted from the analysis. The methylation score for each CpG was represented as a value (β) between 0 (non-methylated) and 1 (completely methylated) according to the fluorescent intensity ratio. Methylation probes covering CpGs within proximal promoters were primarily used. These were defined as CpG sites located within 1,500 bp upstream of the described transcription start site, in the 5’ UTR or first exon [80]. Thus, probes residing within the 3’ UTR or gene body were excluded. Whenever a miRNA had designated probes (consulting the annotation file), these were used. Out of the 575 miRNA loci considered, 448 had designated probes. For miRNAs without designated probes and residing within the introns of coding host genes (‘intronic miRNAs’), the host gene CpG promoter probes were used (63 miRNA loci). For all remaining miRNA loci, the nearest probe was used as an approximation (64 miRNA loci). The methylation probe extractions resulted in 2,587 probes representing the 575 miRNA promoter loci (Additional file 10). When more than one probe represented a miRNA, the median β-value was used. To call aberrations in methylation status, the following method was used: for each miRNA, the median (*m*) and the standard deviation (SD) of β for the normal samples were calculated. Next, the miRNA was called as hypermethylated in a tumor sample if β exceeded *m +* (2 × SD), and as hypomethylated if β was less than *m* - (2 × SD).

#### Pyrosequencing

The methylation status of 18 selected CpG sites for which the genomic positions were represented by a probe on the HumanMethylation450 BeadChip microarray were chosen for replication by pyrosequencing in the replication cohort [81]. These CpG sites were located in the promoters (within 1,500 bp before the transcription start site) corresponding to 26 miRNAs (Additional file 11) that were selected for replication due to their association with methylation alteration in the discovery cohort. DNA (500 ng) isolated from tumor tissue of 122 patients of the DBCG patient cohort and normal breast tissue samples from 15 mammoplastic reductions [78] were bisulfite treated using the EpiTect Fast DNA Bisulfite Kit (Qiagen). One tumor sample did not have DNA left and was therefore excluded from the pyrosequencing analysis. The bisulfite converted DNA was eluted in 15 μl and diluted to a total volume of 70 μl. The PyroMark PCR Kit reagents (Qiagen) were used in the PCR reactions and the pyrosequencing was carried out on a PyroMark Q96 MD system with the PyroMark Gold Q96 Reagents (Qiagen). The quantitative DNA methylation results were analyzed in the Q-CpG software (V.1.0.9, Biotage, Qiagen). The PCR and sequencing primers and the PCR and sequencing conditions are specified in Additional file 11 together with the percentage methylation output per CpG site. The methylation percentage per CpG site was scored as hypo- or hypermethylated using the methylation percentage of the normal samples as a reference defining the aberration threshold similarly as for the discovery cohort using *m ±* (2 × SD) as aberration cut-off. For one assay, cg09918657 (assay 13), the standard deviation among the normal was quite high and the cut-off value for determining samples as hypomethylated was negative and was thus not assessed. Another assay, cg27388703 (assay 16), did not score any samples as altered. See Additional file 11 for details.

### DNA copy number analysis

The DNA copy number data for the discovery cohort were generated using Illumina Human-1 109 k BeadChip SNP arrays (Illumina) and are described in [82]. For the replication cohort, the Comparative Genomic Hybridization 244 k Agilent Microarrays (Agilent Technologies) were used [83]. Copy number data were log_2_-transformed and centered, and for each sample a segmentation was performed using the Piecewise Constant Fitting (PCF) algorithm implemented in the Bioconductor R package *Copynumber*[84]. The average log-transformed copy number was then calculated for each segment and assigned to each probe in the segment. The trade-off between sensitivity and specificity was set to the default value (γ = 40) in the discovery dataset and slightly larger (γ = 50) in the replication dataset to take into account the array resolution (as recommended by the software instructions). In order to assign a copy number to each miRNA locus, the segment copy number average found to cover the miRNA was used to represent the miRNA copy number. For the discovery cohort the miRNA annotation was for this purpose converted from the hg19 to the hg17 build by using the liftOver tool in the UCSC Genome Browser [85]. Copy number aberrations were called by defining miRNAs with segmented copy number values above 0.1 as gains, and values below -0.1 as losses.

### mRNA expression profiling

The mRNA expression data for the discovery dataset were measured using Agilent 4x44K one-color oligonucleotide arrays (Agilent Technologies) and have previously been published [4] and submitted to GEO with accession number GSE19783. For the replication dataset the mRNA was measured using the Human Genome Survey Microarray version 2.0 (Applied Biosystems), and the data were submitted to GEO with accession number GSE24117 [75,83].

### Identification of in-*cis* miRNAs

#### Discovery cohort

miRNAs altered by either copy number or DNA methylation level in*-cis* were referred to as in-*cis* miRNAs. To identify in-*cis* miRNAs associated with hypomethylation or gain, each miRNA in each patient was assigned to one of the two groups ‘altered’ or ‘non-altered’ based on (i) copy number and (ii) DNA methylation. A Wilcoxon rank-sum test was performed for each miRNA to assess whether the miRNA expression was significantly different in the two groups of altered and non-altered patients. The resulting *P*-values were corrected for multiple comparison using the Benjamini-Hochberg FDR [86]. A FDR-corrected *P*-value < 0.05 was considered as statistically significant. Three tests were performed: (1) considering aberrations on the copy number level or (2) the methylation level, or (3) both. The latter category contained samples with at least one aberration at either the copy number or methylation level, whereas patients with no aberrations were in the alternative group. To identify miRNAs associated with hypermethylation or loss, an analogous procedure was used. The statistically significant miRNAs were visually inspected by graphing the miRNA expression as a function of copy number or methylation level, and only miRNAs with positive correlation between copy number and expression or negative correlation between methylation level and expression were further considered.

#### Replication cohort

As genome-wide copy number was available for the replication cohort and pyrosequencing was only performed for a selection of miRNAs, the replication of the in-*cis* miRNAs was performed separately for the two aberration types. Of the 70 in-*cis* miRNAs, 69 were present in the replication cohort and thus targets of replication (miR-624-5p was not expressed in the replication cohort). The expression of the 69 in-*cis* miRNAs was tested for association with copy number alterations by the same approach as for the discovery cohort. Similarly, the expression of the 26 miRNAs selected for pyrosequencing replication was tested for differential expression after grouping the tumor samples into altered or non-altered groups based on percentage methylation per CpG site and using the normal samples as a reference. Among the 18 CpG sites, four probes represented two CpG sites in the same promoter area (cg07641807 and cg23665802 on chromosome 13 for assays 11 and 12, and cg00057966 and cg10530767 on chromosome 17 for assays 14 and 15, respectively). For these instances, a Wilcoxon rank-sum test was performed separately, attaining the highest *P*-values. In-*cis* miRNAs that were consistently statistically significant (*P* < 0.05) in the discovery and replication cohorts were considered replicated.

### Test for differential miRNA expression in clinical and molecular subgroups

To investigate differential expression of the identified in*-cis* miRNAs in clinical subgroups and molecular subgroups, non-parametric tests were applied. The Wilcoxon rank-sum test was used for two-group comparison and the Kruskal-Wallis one-way analysis of variance for three-group comparison (histological grade). A significance level of *P* < 0.05 was chosen after FDR correction. A log-rank test was used for statistical comparison of Kaplan-Meier survival curves (3 of the 89 samples in the discovery cohort did not have survival data and were thus excluded from this analysis).

### Cell culture

MCF-7 cells [87,88] were purchased from Interlab Cell Line Collection (ICLC, Genova, Italy) and cultured in DMEM (1 g/l glucose; Sigma-Aldrich, St Louis, MO, USA) supplemented with 10% fetal bovine serum (FBS), 2 mM L-glutamine and 1% penicillin/streptomycin. JIMT-1 cells [89] were obtained from The German Collection of Microorganisms and Cell Cultures (DSMZ, Leibniz, Germany), and they were cultured in 1:1 Ham's F-12/DMEM (4.5 g/l glucose) supplemented with 10% FBS, 10 μg/ml insulin, 2 mM L-glutamine and 1% penicillin/streptomycin. KPL-4 cells [90] were a kind gift from Prof. Junichi Kurebayashi (Kawasaki Medical School, Japan), and they were cultured in DMEM (4.5 g/l glucose) supplemented with 10% FBS, 2 mM L-glutamine and 1% penicillin/streptomycin.

### miRNA functional assays

For the functional assays, cells were transfected with Dharmacon miRIDIAN microRNA mimics (20 nM; Dharmacon, Lafayette, CO, USA) in 384-well plates using SilentFect (Bio-Rad Laboratories, Hercules, CA, USA) as described previously [9,91]. After 72 h incubation, cell viability was assayed by CellTiter-GLO cell viability assay (Promega Corp., Madison, WI, USA). The results were Loess normalized [92] and log_2_-transformed. Values ±2 × SD, were considered as significant, which corresponded to a threshold of |0.2|.

For protein lysate microarray analysis, cells were lysed 72 h after transfection and printed on nitrocellulose-coated microarray FAST™ slides (Whatman Inc., Florham Park, NJ, USA). Ki67, cPARP and p-AKT were detected by staining the slides with Ki67 antibody (#M7240, Dako, Glostrup, Denmark), cPARP antibody (#ab32064, Abcam, Cambridge, UK), and p-AKT(S473) antibody (#9271, Cell Signaling Technology Inc., Danvers, MA, USA), respectively, followed by exposure to Alexa Fluor 680-tagged secondary antibodies (Invitrogen Inc.). For total protein measurement, the arrays were stained with Sypro Ruby Blot solution (Invitrogen Inc.). The slides were scanned with Tecan LS400 (Tecan Inc., Durham, NC, USA) microarray scanner and Odyssey Licor IR-scanner (LI-COR Biosciences, Lincoln, NE, USA) to detect the Sypro, Ki67, cPARP, and p-AKT signals. Array-Pro Analyzer microarray analysis software (Median Cybernetics Inc., Bethesda, MD, USA) was used for analyzing the data. The lysate microarray data were log_2_-transformed and converted into z-scores by subtracting the mean of the whole screen and dividing by the standard deviation of the whole screen. Values ±2 × SD were considered as significant, which corresponded to a threshold of |1.96|.

### Network analysis using IPA

Networks containing mRNAs correlated to candidate miRNAs were generated with IPA (Ingenuity Systems) [93]. For each candidate miRNA, a dataset containing the list of all the positively correlated (Spearman’s rho >0.3) and negatively correlated (Spearman’s rho < -0.3) mRNAs (overlapping both cohorts) was uploaded into the application. Each identifier was mapped to its corresponding object in the Ingenuity Knowledge Base. These molecules were overlaid onto a global molecular network developed from information contained in the Ingenuity Knowledge Base, and networks were then algorithmically generated based on their connectivity. The confidence level was chosen to include ‘experimentally observed’ and ‘high (predicted)’ relations.

### mRNA co-expression analysis using SEEK

SEEK [94] is a novel meta-analysis tool that incorporates a large human microarray expression compendium. It takes a set of gene identifiers as input and finds genes that are co-expressed in the compendium with the input genes. We first utilized SEEK to determine the extent to which the mRNAs that we identified as positively or negatively correlated to each candidate miRNA were supported by other datasets. We input the list of correlated mRNAs as query. The top 50 datasets related to the query were dominated by breast cancer and included the mRNA dataset we used in our analysis. We next retrieved the co-expressed neighbors of the query genes, and performed gene-set enrichment analysis on the neighbor set. By selecting these genes rather than the query genes for enrichment, we utilize the robustness due to the result of integrating many microarray datasets. For the enrichment analysis, the top 1,000 genes surrounding the query were analyzed for overrepresentation in each of 1,371 Gene Ontology biological processes (with human annotations). Processes with a FDR-corrected *P*-value < 0.05 were considered statistically significant.

## Abbreviations

bp: Base pair; cPARP: Cleaved poly (ADP-ribose) polymerase; DBCG: Danish Breast Cancer Cooperative Group; DMEM: Dulbecco’s modified Eagle medium; ER: Estrogen receptor; FBS: Fetal bovine serum; FDR: False discovery rate; GEO: Gene expression omnibus; HER2: Human epidermal growth factor receptor 2; IPA: Ingenuity pathway analysis; m: Median; miRNA: microRNA; p-AKT: Phosphorylated AKT; SD: Standard deviation; SEEK: Search-based exploration of expression compendium; UTR: Untranslated region.

## Competing interests

The authors declare that they have no competing interests.

## Authors’ contributions

Conceived and designed the approach: MRA, QZ, RL, SH, OGT and OCL. Conceived and designed the experiments: SKL, TF, GIGA, MP, FB, JT, ALBD, KKS and VNK. Analyzed the data: MRA, SKL, TF, QZ, NT, JT, ALBD, OCL, KKS and VNK. Interpreted the results: MRA, SKL, TF and RL. Provided breast cancer samples and clinical information: JO, JA and TT. Wrote the paper: MRA, KKS and VNK. All authors read and approved the final manuscript.

## Supplementary Material

Additional file 1 **Overview of the 70 in*-cis *miRNAs identified in the discovery cohort.** For each of the in*-cis* miRNAs, miRNA name, MIMAT id, chromosomal location, copy number-miRNA expression correlation and methylation-miRNA expression correlation with *P*-values and aberration type are listed. The miRNAs in bold font are those that were replicated in an independent cohort. Click here for file

Additional file 2 **Outline of the approach used to identify in-*cis *miRNAs.** The genomic locus (or loci) of all expressed miRNAs were identified, and each sample was scored as altered or non-altered with respect to DNA methylation status and copy number. Wilcoxon rank-sum tests were applied to test whether alterations at the copy number or methylation levels were associated with miRNA expression. In the discovery cohort, 70 in-*cis* miRNAs were identified. Of these, 41 were replicated in an independent cohort. *Accounting for the possibility that one mature miRNA may have more than one genomic origin; this corresponds to 461 unique miRNAs. Click here for file

Additional file 3 **Aberration pattern of miRNA family members across the patients.** The aberration pattern of 13 miRNAs that are members of the five families listed in Table 1 are shown for the patients in the discovery cohort (n = 89; only aberrations of the type hypomethylation or gain are shown). miRNA family name, miRNA ids and genome coordinates are listed. The color coding represents aberration type for single patients (see legend under the table). One ‘Overall result’ row is indicated per miRNA family, which represents the total activating aberration state of a patient for that miRNA family. Click here for file

Additional file 4 **Scatterplots comparing copy number-expression and methylation-expression correlation in the discovery and replication cohort. (a)** Scatterplot representing correlation between copy number and miRNA expression for the 69 in*-cis* miRNAs in the discovery and replication cohorts (one in-*cis* miRNA was not expressed in the replication cohort). **(b)** Scatterplot representing correlation between methylation status and miRNA expression in the discovery and replication cohorts for the 26 miRNAs assessed by pyrosequencing in the replication cohort. Black circles represent miRNAs that were initially identified in the discovery cohort and later confirmed in the replication cohort (with respect to aberration type and association to expression). Open circles represent miRNAs that were not confirmed in the replication cohort. Corr, Spearman correlation coefficient. Click here for file

Additional file 5 **Replicated miRNAs and association with clinical parameters (in at least one cohort).** Shown are all of the replicated in-*cis* miRNAs that were found significantly differentially expressed between clinical or molecular subtypes in at least one of the two cohorts. Significant *P*-values are highlighted in bold (*P* < 0.05). Click here for file

Additional file 6 **Boxplots showing miRNA expression within patient aberration groups for four candidate miRNAs. (a)** miR-21-3p expression in the discovery and replication cohort. **(b)** miR-151a-5p expression in the discovery and replication cohort. **(c)** miR-148b-3p expression in the discovery and replication cohort. **(d)** let-7e-3p expression in the discovery and replication cohort. The *P*-values are from Wilcoxon rank-sum tests. Hypermet, hypermethylated. Click here for file

Additional file 7 **Genes correlated to candidate miRNAs.** For each of the four candidate miRNAs, the genes that were consistently found correlated (miRNA-mRNA Spearman correlation > |0.3|) in both the discovery and replication cohorts are listed. The genes are sorted by increasing correlation based on the discovery cohort’s correlation values. Click here for file

Additional file 8 **Enrichment of genes co-expressed with correlated genes.** For each of the four candidate miRNAs, the positively or negatively correlated genes were used as query input to the SEEK tool [94]. The set of co-expressed genes thus identified was used for functional enrichment analysis. Gene Ontology biological process terms that were found enriched among the co-expressed genes are listed together with the FDR-corrected *P*-values. Click here for file

Additional file 9 **Networks of genes correlated to the four candidate miRNAs.** The list of correlated genes was imported into IPA, and networks were generated based on known and predicted associations and interactions of the corresponding proteins. Solid lines represent direct relationships and dotted lines represent indirect relationships. Proteins colored in red are positively correlated to the miRNA (Spearman’s rho >0.3), and proteins colored in green are negatively correlated (Spearman’s rho < -0.3). White proteins are not among the genes found correlated to a miRNA. The legend shows protein function. Network of proteins (genes) correlated to **(a)** miR-148b-3p, **(b)** let-7e-3p and **(c)** miR-21-3p. The networks were generated through the use of IPA (Ingenuity® Systems [93]). Click here for file

Additional file 10 **Discovery cohort data (575 miRNAs × 89 patients).** The miRNA copy number, methylation and expression data analyzed for the discovery cohort are provided in different sheets. Click here for file

Additional file 11 **Replication cohort data (69 miRNAs × 123 patients).** The miRNA copy number, methylation and expression data analyzed for the replication cohort are provided in different sheets. Click here for file
