# Supplementary figures and images for: Individual and combined effects of DNA methylation and copy number alterations on miRNA expression in breast tumors
Source: Genome Biol. 2013 Nov 20;14(11):R126. doi: 10.1186/gb-2013-14-11-r126 (PMC4053776; doi:10.1186/gb-2013-14-11-r126)

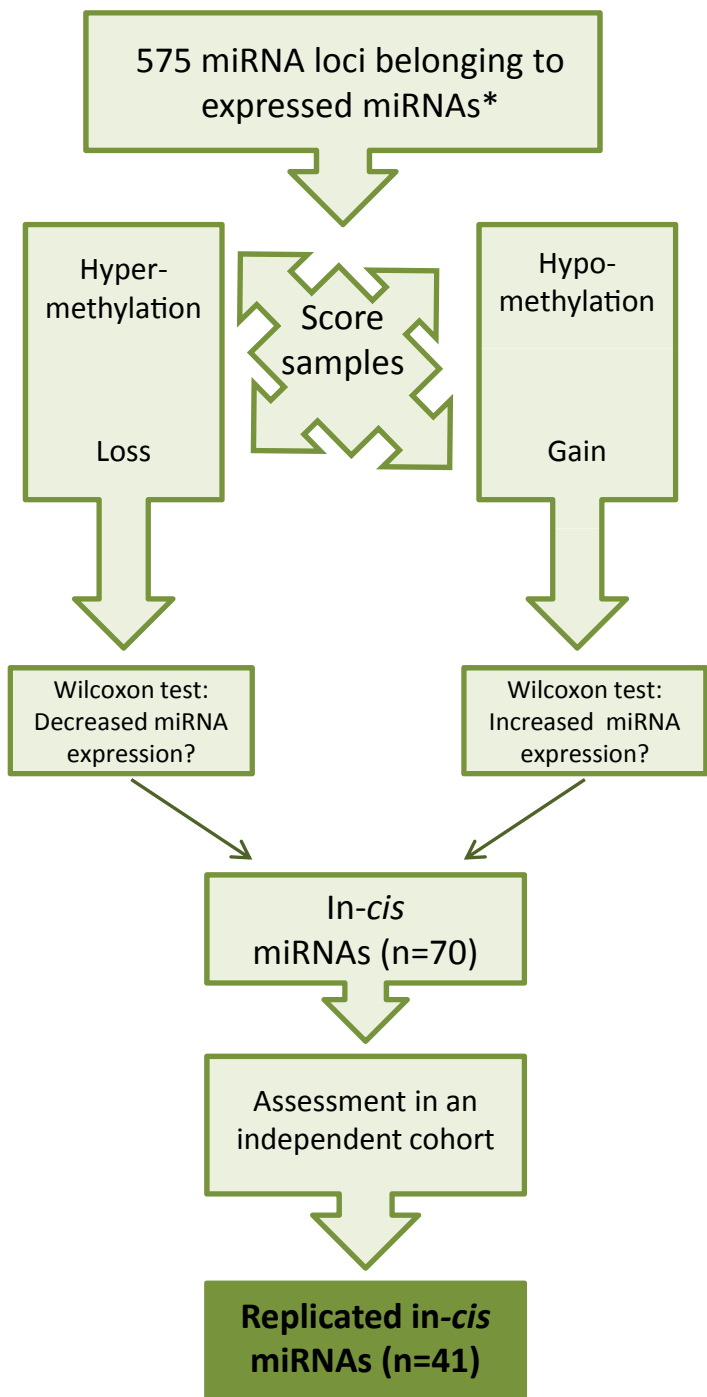

Supplement: Additional file 2 — Outline of the approach used to identify in-cis miRNAs. The genomic locus (or loci) of all expressed miRNAs were identified, and each sample was scored as altered or non-altered with respect to DNA methylation status and copy number. Wilcoxon rank-sum tests were applied to test whether alterations at the copy number or methylation levels were associated with miRNA expression. In the discovery cohort, 70 in-cis miRNAs were identified. Of these, 41 were replicated in an independent cohort. *Accounting for the possibility that one mature miRNA may have more than one genomic origin; this corresponds to 461 unique miRNAs. [file gb-2013-14-11-r126-S2.pdf]

(a)

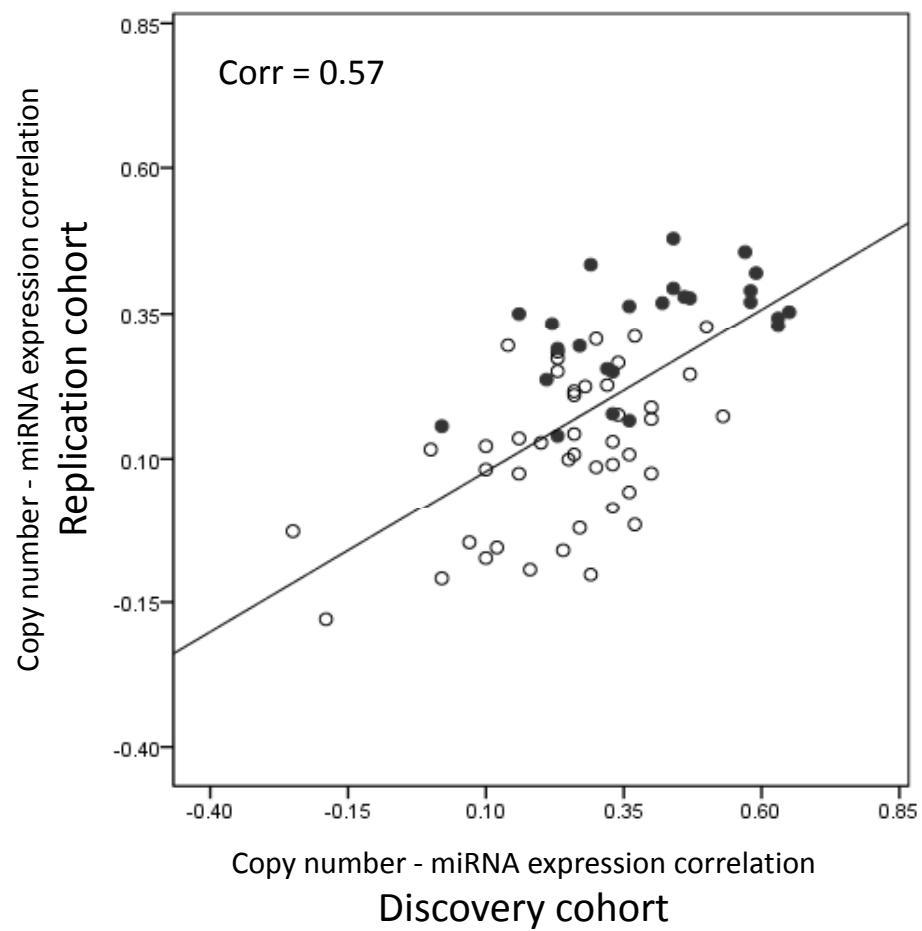

(b)

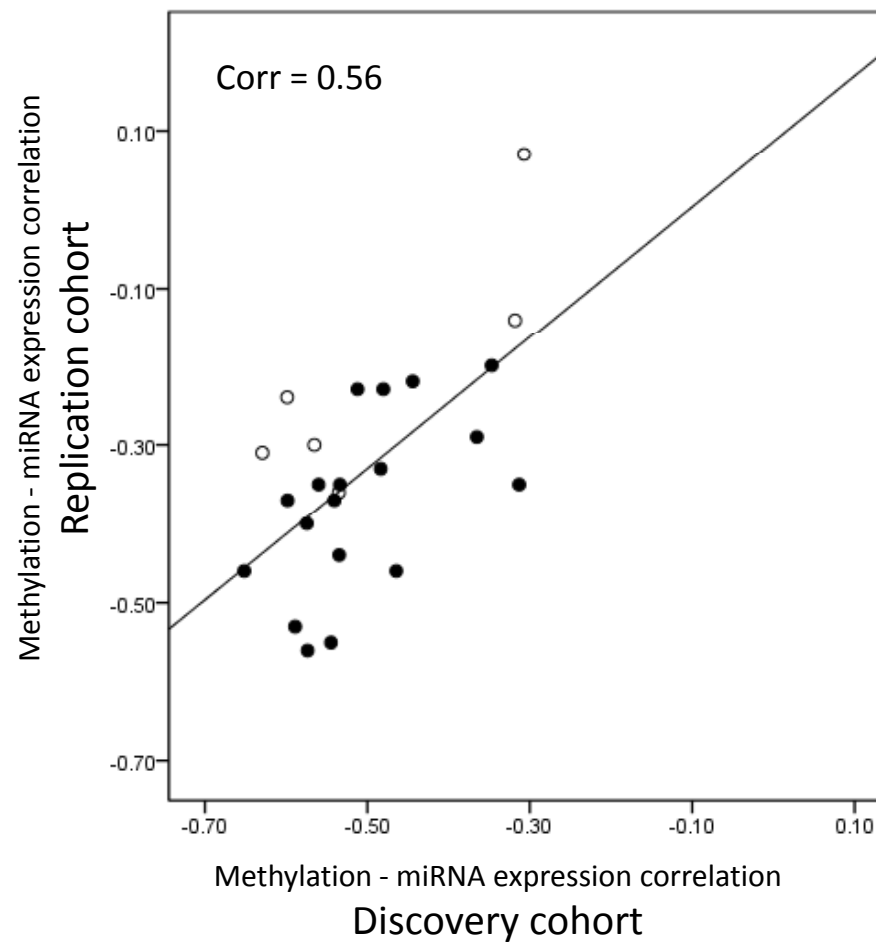

- Confirmed in replication cohort
- Not confirmed in replication cohort

Supplement: Additional file 4 — Scatterplots comparing copy number-expression and methylation-expression correlation in the discovery and replication cohort. (a) Scatterplot representing correlation between copy number and miRNA expression for the 69 in-cis miRNAs in the discovery and replication cohorts (one in-cis miRNA was not expressed in the replication cohort). (b) Scatterplot representing correlation between methylation status and miRNA expression in the discovery and replication cohorts for the 26 miRNAs assessed by pyrosequencing in the replication cohort. Black circles represent miRNAs that were initially identified in the discovery cohort and later confirmed in the replication cohort (with respect to aberration type and association to expression). Open circles represent miRNAs that were not confirmed in the replication cohort. Corr, Spearman correlation coefficient. [file gb-2013-14-11-r126-S4.pdf]

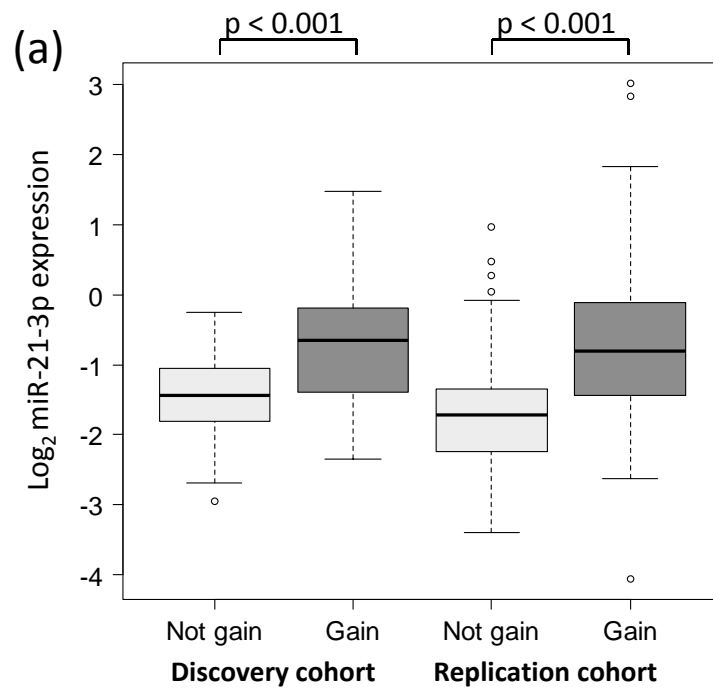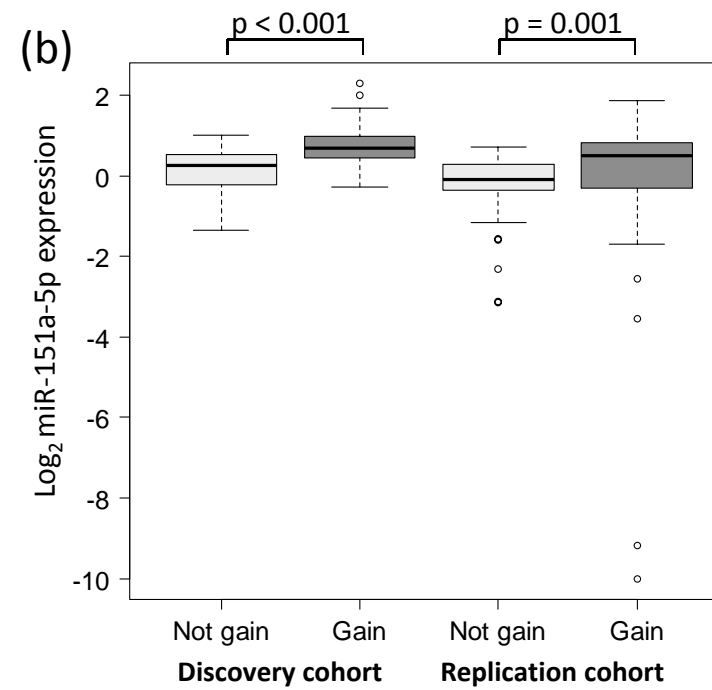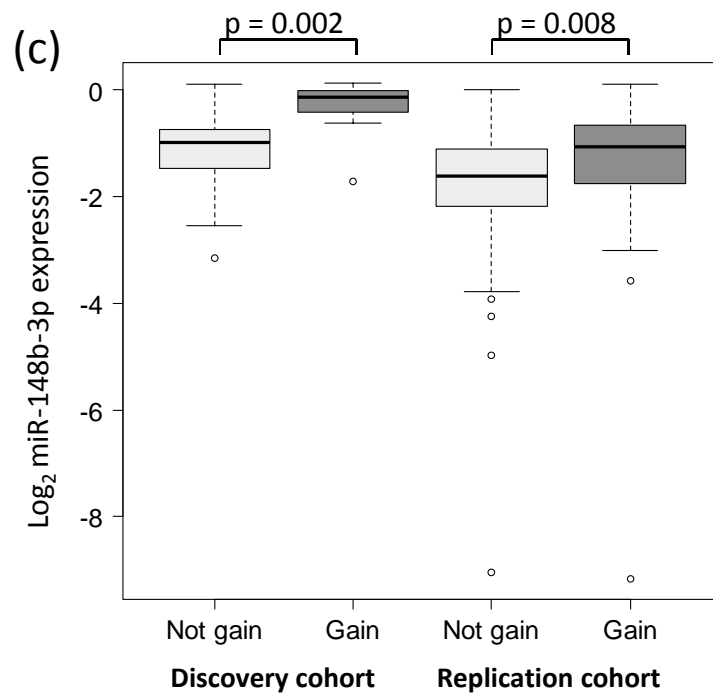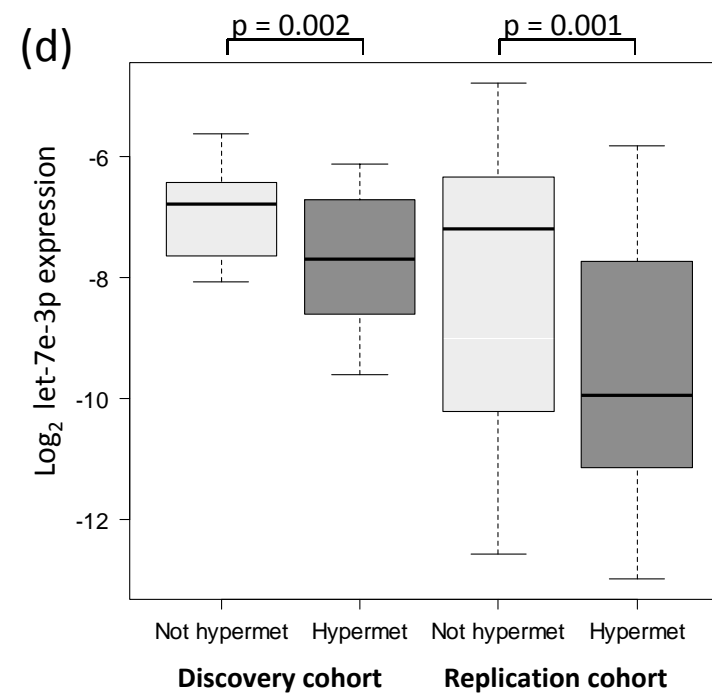

Supplement: Additional file 6 — Boxplots showing miRNA expression within patient aberration groups for four candidate miRNAs. (a) miR-21-3p expression in the discovery and replication cohort. (b) miR-151a-5p expression in the discovery and replication cohort. (c) miR-148b-3p expression in the discovery and replication cohort. (d) let-7e-3p expression in the discovery and replication cohort. The P-values are from Wilcoxon rank-sum tests. Hypermet, hypermethylated. [file gb-2013-14-11-r126-S6.pdf]

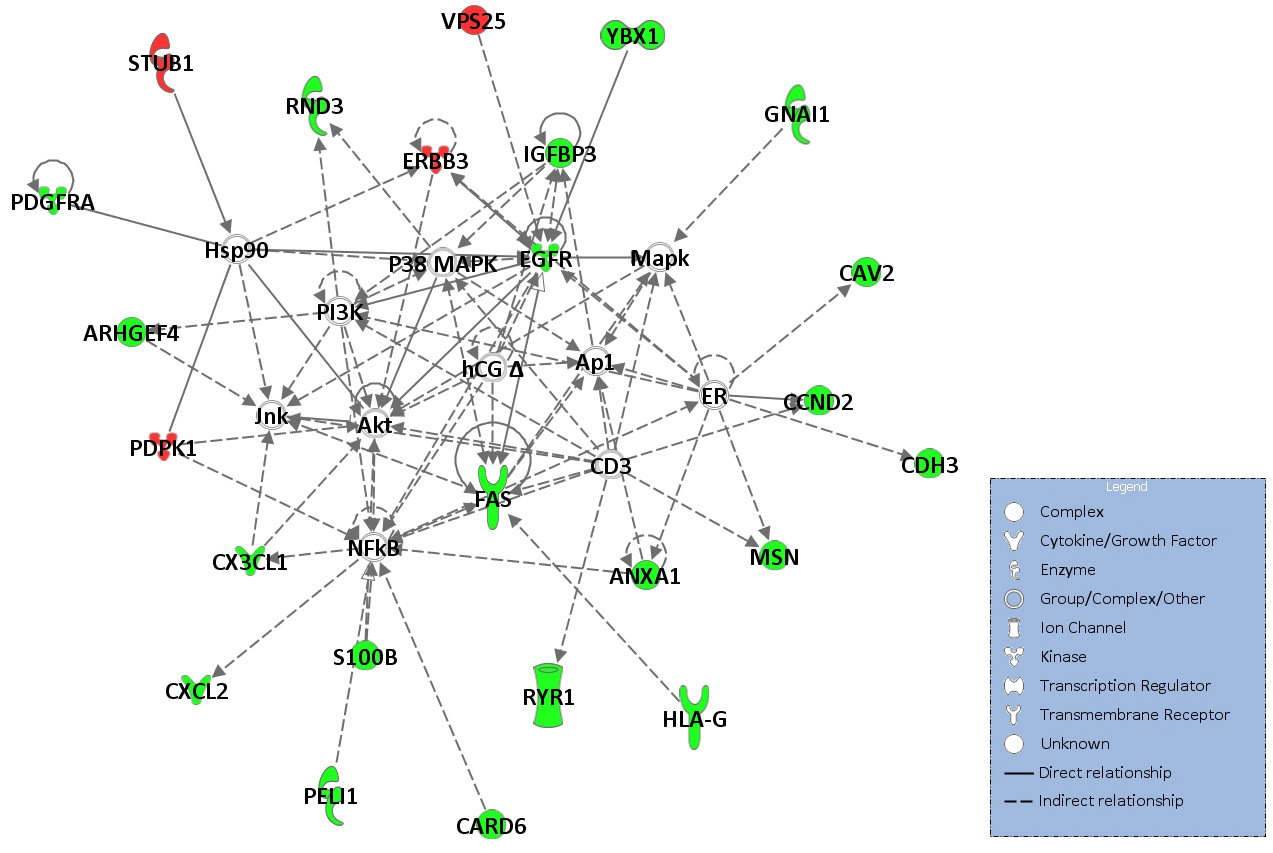

Supplement: Additional file 9 — Networks of genes correlated to the four candidate miRNAs. The list of correlated genes was imported into IPA, and networks were generated based on known and predicted associations and interactions of the corresponding proteins. Solid lines represent direct relationships and dotted lines represent indirect relationships. Proteins colored in red are positively correlated to the miRNA (Spearman’s rho >0.3), and proteins colored in green are negatively correlated (Spearman’s rho < -0.3). White proteins are not among the genes found correlated to a miRNA. The legend shows protein function. Network of proteins (genes) correlated to (a) miR-148b-3p, (b) let-7e-3p and (c) miR-21-3p. The networks were generated through the use of IPA (Ingenuity® Systems [93]). [file gb-2013-14-11-r126-S9.zip › Additional file 9a.jpeg]

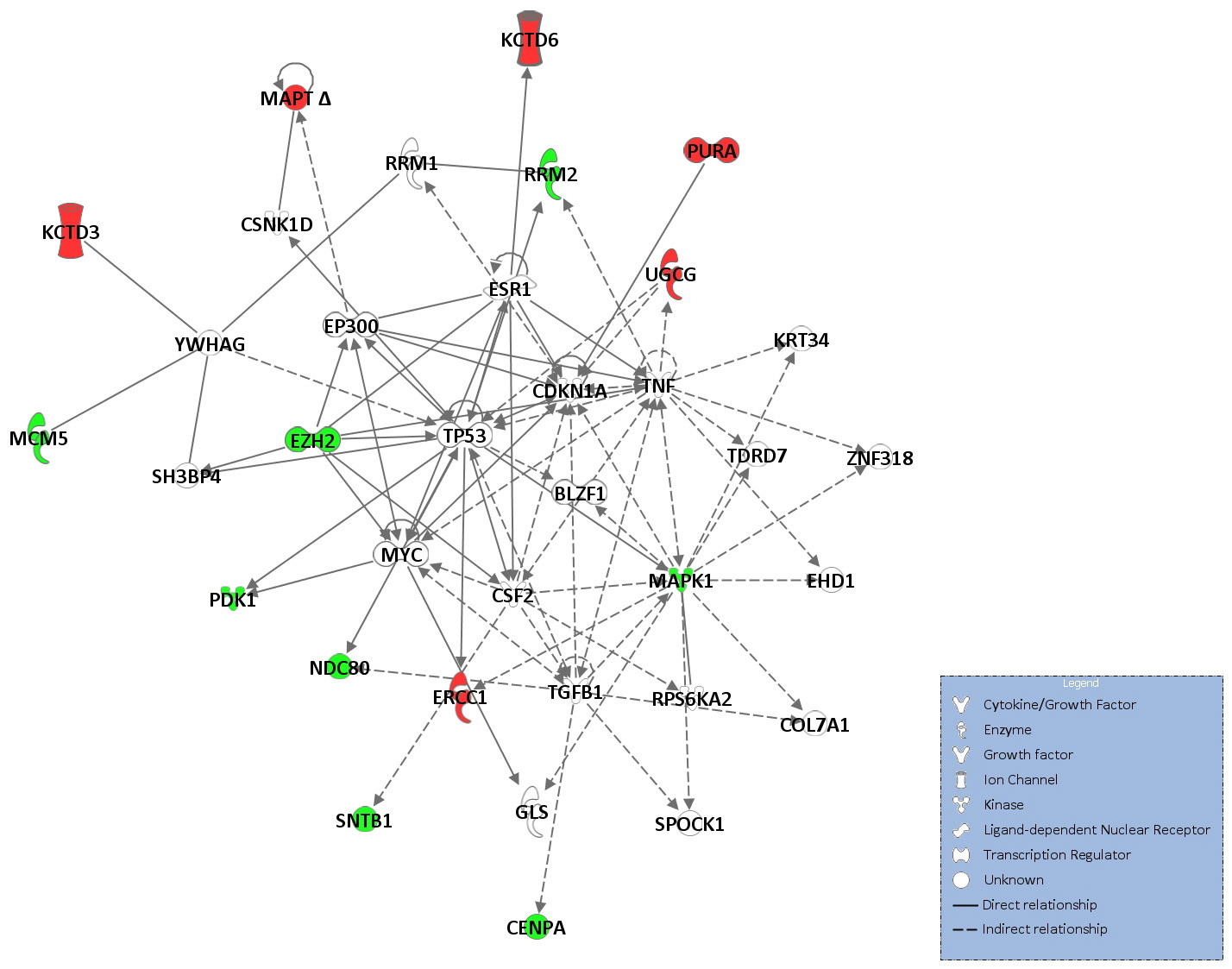

Supplement: Additional file 9 — Networks of genes correlated to the four candidate miRNAs. The list of correlated genes was imported into IPA, and networks were generated based on known and predicted associations and interactions of the corresponding proteins. Solid lines represent direct relationships and dotted lines represent indirect relationships. Proteins colored in red are positively correlated to the miRNA (Spearman’s rho >0.3), and proteins colored in green are negatively correlated (Spearman’s rho < -0.3). White proteins are not among the genes found correlated to a miRNA. The legend shows protein function. Network of proteins (genes) correlated to (a) miR-148b-3p, (b) let-7e-3p and (c) miR-21-3p. The networks were generated through the use of IPA (Ingenuity® Systems [93]). [file gb-2013-14-11-r126-S9.zip › Additional file 9b.jpeg]

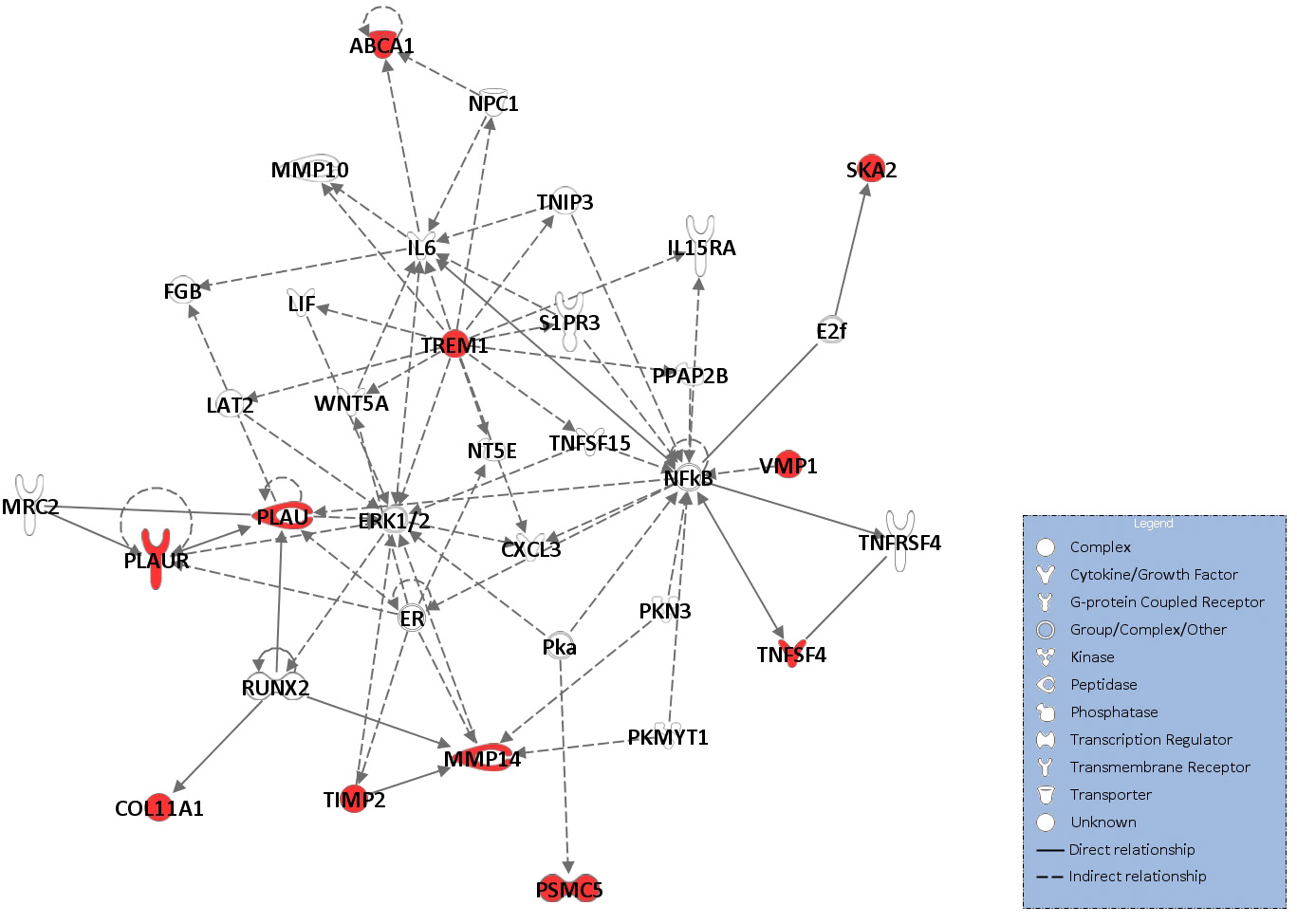

Supplement: Additional file 9 — Networks of genes correlated to the four candidate miRNAs. The list of correlated genes was imported into IPA, and networks were generated based on known and predicted associations and interactions of the corresponding proteins. Solid lines represent direct relationships and dotted lines represent indirect relationships. Proteins colored in red are positively correlated to the miRNA (Spearman’s rho >0.3), and proteins colored in green are negatively correlated (Spearman’s rho < -0.3). White proteins are not among the genes found correlated to a miRNA. The legend shows protein function. Network of proteins (genes) correlated to (a) miR-148b-3p, (b) let-7e-3p and (c) miR-21-3p. The networks were generated through the use of IPA (Ingenuity® Systems [93]). [file gb-2013-14-11-r126-S9.zip › Additional file 9c.jpeg]
